# Microplastic toxicity in marine microalgae: an ecotoxicological synthesis of experimental biological responses

**DOI:** 10.1007/s10646-026-03181-x

**Published:** 2026-10-01

**Authors:** Nágela Gardênia Rodrigues Santos, Késsia Fernanda Brito Pinho, Itala de Cassia Sousa Reis, Ricardo Luvizotto-Santos, Marianna Basso Jorge

**Affiliations:** 1 Aquatic Ecotoxicology Group and Postgraduate Program in Biodiversity and Biotechnology, Federal University of Maranhão (UFMA), São Luís, MA Brazil; 2 Aquatic Ecotoxicology Group, Federal University of Maranhão (UFMA), São Luís, MA Brazil; 3 Aquatic Ecotoxicology Group and Postgraduate Program in Environmental Science and Technology, Federal University of Maranhão (UFMA), São Luís, MA Brazil; 4 Aquatic Ecotoxicology Group, Postgraduate Program in Environmental Science and Technology and Postgraduate Program in Biodiversity and Biotechnology, Federal University of Maranhão (UFMA), São Luís, MA Brazil

**Keywords:** Primary producers, Oxidative stress, Photosynthesis, Emerging contaminants, Environmental risk

## Abstract

As a pervasive form of marine pollution, microplastics (MPs) are widely distributed across coastal and oceanic systems, raising concerns about their ecological impacts. This review provides an evidence-based synthesis of microplastic–microalgae interactions, integrating physiological responses and associated toxicity pathways. Marine microalgae are particularly relevant in this context because they constitute the foundation of marine food webs and play a central role in primary productivity and global biogeochemical cycling. This study synthesizes experimental ecotoxicological evidence on the effects of microplastics on marine microalgae, with the aim of identifying dominant toxicity endpoints and key knowledge gaps relevant to ecotoxicological evaluation. Publications indexed in Web of Science and Scopus from 1970 to August 2025 were systematically screened, resulting in 59 selected publications, of which 43 experimental studies met the criteria for the comparative ecotoxicological synthesis. The analysis revealed a rapid expansion of research on microplastic–microalgae interactions, with experimental studies predominantly investigating polystyrene and polyethylene, across a broad range of particle sizes. The most frequently investigated biological responses included growth/population parameters, cellular stress and damage indicators, and photosynthetic responses. Despite this progress, several ecologically relevant endpoints and processes remain insufficiently investigated, including aggregate formation, particle sinking dynamics, DNA damage, and long-term exposure under environmentally realistic conditions. Furthermore, most experimental studies rely on short-term laboratory assays using simplified exposure scenarios, which may limit the ecological relevance of current knowledge. Taken together, the available evidence highlights key research priorities and outlines directions for improving experimental ecotoxicological studies.

**Supplementary Information:**

The online version contains supplementary material available at https://doi.org/10.1007/s10646-026-03181-x.

## Introduction

Microplastics (MPs) have emerged as pervasive environmental contaminants due to their widespread occurrence across aquatic, terrestrial, and atmospheric compartments (Nava and Leoni 2021). Over recent decades, exponential growth in plastic production and consumption has intensified the release of plastic debris into the environment; global plastics production doubled between 2000 and 2019, increasing from 234 to 460 million tonnes (OECD 2022). This plastic debris undergoes continuous fragmentation and persists for extended periods, resisting physical, chemical, and biological degradation (Cole et al. 2011). Consequently, MPs have become globally distributed pollutants, raising significant concerns regarding their ecological implications (Wang et al. 2025b). In marine environments, microplastics are now recognized as a major component of anthropogenic pollution, affecting ecosystem structure and function across multiple spatial scales.

Microplastics are generally defined as plastic particles ranging from 1 μm to 5 mm and may originate either as primary particles intentionally manufactured at microscopic scales or as secondary particles generated through the fragmentation and weathering of larger plastic debris (Andrady 2011, 2017; Hernandez et al. 2017). They occur in diverse shapes and polymer compositions, including polyethylene (PE), polypropylene (PP), polystyrene (PS), polyvinyl chloride (PVC), and polyethylene terephthalate (PET). Their size, shape, polymer composition, and surface properties influence environmental behavior, bioavailability, and interactions with organisms and co-occurring contaminants (Cole et al. 2011; Wright et al. 2013).

The occurrence of MPs has been documented in virtually all aquatic environments, including rivers, lakes, estuaries, coastal zones, and open oceans, with concentrations fluctuating according to geographic location, hydrodynamics, and anthropogenic pressure (Horton et al. 2017). In marine systems, MPs are recognized not only as physical stressors but also as dynamic vectors capable of adsorbing and transporting metals, persistent organic pollutants (POPs), pharmaceuticals, and other emerging contaminants (Rochman et al. 2013). This multifunctional role reinforces their classification as complex environmental stressors that can alter the fate and transport of contaminants across environmental compartments (Koelmans et al. 2016).

As the abundance of MPs increases, so does their bioavailability to marine organisms across multiple trophic levels (Wright et al. 2013). Numerous studies have documented MP ingestion and associated adverse effects in zooplankton, benthic invertebrates, mollusks, crustaceans, and fish (Cole et al. 2013; Lusher et al. 2013). These interactions raise critical questions regarding trophic transfer and broader food-web consequences, particularly under co-exposure to chemical contaminants (Rochman et al. 2013).

Marine microalgae occupy a central position in aquatic ecosystems as primary producers, supporting aquatic food webs, driving biogeochemical cycles, and contributing significantly to global carbon sequestration (Falkowski et al. 1998). Due to their small size, high surface-area-to-volume ratio, and continuous exposure to surrounding water, microalgae are particularly vulnerable to MPs exposure (Bhattacharya et al. 2010). Experimental studies have demonstrated that MPs may impair microalgal growth, photosynthetic activity, cellular morphology, and may induce oxidative stress and alter biochemical composition (Sjollema et al. 2016; Zhang et al. 2017). These effects may arise from physical cell–surface interactions, shading effects that limit light availability, or chemical interactions involving co-occurring contaminants associated with plastic particles (Bhattacharya et al. 2010; Koelmans et al. 2016; Prata et al. 2018).

Such alterations in microalgal physiology have implications extending beyond organism-level toxicity. Changes in primary productivity, photosynthetic efficiency, and biomass production may propagate across food webs, influencing nutrient cycling and essential ecosystem services (Falkowski et al. 1998; Nava and Leoni 2021). Consequently, understanding MPs–microalgae interactions is critical for evaluating the broader environmental risks associated with plastic pollution (SAPEA 2019).

However, despite the rapid expansion of research on microplastic–marine microalgae interactions, the available experimental evidence remains fragmented and heterogeneous, with substantial variation in experimental design, particle characteristics, exposure concentrations, and biological endpoints (Hartmann et al. 2019). Previous reviews have provided important overviews of microplastic effects on microalgae and aquatic ecosystem functioning, but a systematic comparative synthesis focused specifically on the experimental ecotoxicological evidence for marine microalgae remains limited. Consequently, it remains difficult to determine which biological responses are consistently supported across studies, how experimental conditions influence the reported effects, and which methodological limitations constrain environmental extrapolation. Addressing these gaps is important for identifying robust ecotoxicological endpoints and guiding more standardized and environmentally relevant experimental approaches.

To address these gaps, this study provides a comparative synthesis of the experimental ecotoxicological evidence on microplastic effects in marine microalgae, focusing on the most frequently investigated polymer types, particle sizes, exposure conditions, biological endpoints, and reported responses. The literature considered for this synthesis was identified through searches of Web of Science and Scopus covering the period from 1970 to August 2025. In addition, the temporal development of this research field and its main geographic and scientific contributors are described to provide a structured overview of the current state of knowledge. Specifically, this review comparatively evaluates the experimental evidence available for marine microalgae to identify consistent biological responses, methodological limitations, and critical knowledge gaps. Given the ecological role of microalgae as marine primary producers, this synthesis provides a basis for interpreting the ecotoxicological significance of organism-level responses while identifying the limitations that currently constrain extrapolation to environmentally realistic conditions. The resulting evidence framework also supports priorities for more standardized and environmentally relevant experimental approaches.

## Materials and methods

### Data sources and search strategy

A systematic literature search was conducted using two major scientific databases, Web of Science and Scopus. These platforms were selected due to their comprehensive coverage of peer-reviewed environmental and toxicological literature and their frequent use in systematic evidence syntheses (Falagas et al. 2008; Mongeon and Paul-Hus 2016). The combined use of these databases minimizes indexing bias and ensures a more robust evidence base for synthesis (Vieira and Gomes 2009; Donthu et al. 2021).

The search was first performed in February 2024 and updated in August 2025 to include the latest publications in this rapidly evolving field. All retrieved records were exported in BibTeX format for standardized processing (Aria and Cuccurullo 2017). The literature search was conducted using multiple predefined combinations of terms related to microplastics, marine microalgae, toxicity, and marine pollution. In Scopus, searches were performed using the TITLE-ABS-KEY field, whereas in Web of Science the search terms were applied to the Topic field, which includes Title, Abstract, Author Keywords, and Keywords Plus. This approach enabled broader and more comparable coverage across the two databases. The complete database-specific search expressions, search dates, and retrieval counts are provided in Supplementary Table S3.

### Inclusion and exclusion criteria

To ensure relevance and quality, the following inclusion criteria were defined following established bibliometric and evidence-synthesis approaches (Donthu et al. 2021): (i) publications indexed in peer-reviewed journals within the “Environmental Science” and “Toxicology” categories; (ii) a timeframe spanning 1970 to August 2025. The broad starting date was intentionally adopted to ensure comprehensive retrieval and to determine when experimental evidence on *microplastic effects on marine microalgae first emerged in the scientific literature*,* rather than assuming an a priori starting* point for this field; (iii) articles published in English; and (iv) original research articles and review papers.

Conversely, book chapters, conference proceedings, abstracts, data papers, editorials, or gray literature were excluded to maintain citation-based consistency (Falagas et al. 2008). Studies focusing exclusively on freshwater systems, sediments, or non-microalgal taxa were also excluded to preserve thematic focus. Original research articles and review papers addressing microplastic–marine microalgae interactions were retained for the bibliometric characterization of the field. For the comparative ecotoxicological synthesis, however, only original studies reporting experimental exposure to microplastic particles and evaluating biological responses in marine microalgae were considered.

### Screening procedure and data selection

The selection followed a multi-stage screening process consistent with established bibliometric and evidence-synthesis approaches (Zupic and Čater 2015; Donthu et al. 2021). After compiling records from both databases, duplicates were removed using Digital Object Identifiers (DOIs), author names, and publication titles. Titles, abstracts, and keywords were then individually screened for relevance. Articles that did not specifically address interactions between MPs and marine microalgae were excluded. The final dataset comprised 59 publications addressing microplastic–marine microalgae interactions, which were retained for the bibliometric characterization of the field. The complete list of these publications is provided in Supplementary Table S1.

**Fig. 1:**
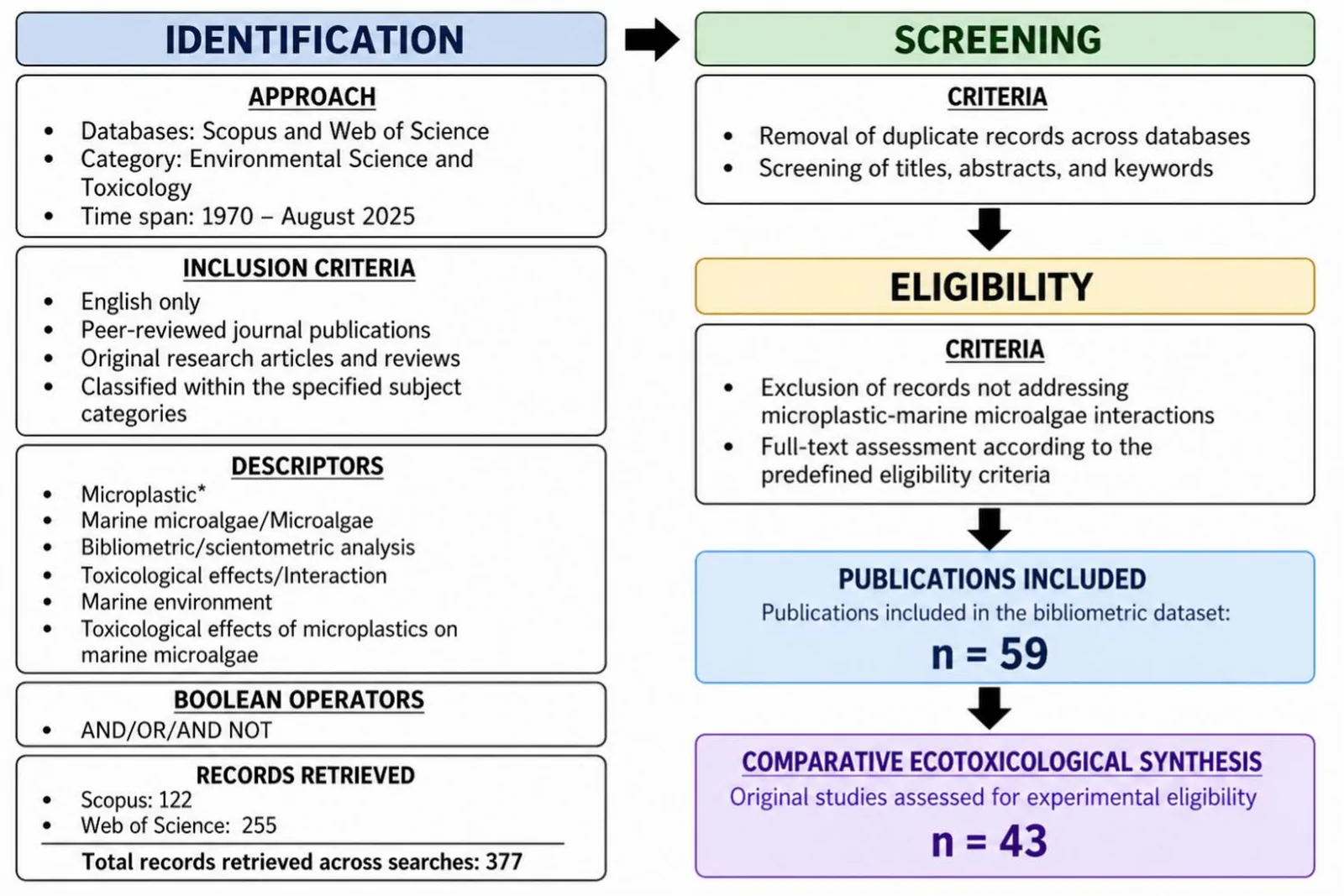
Workflow of the literature search, screening, and selection process for studies addressing microplastic–microalgae interactions

For the comparative ecotoxicological synthesis, these publications were further assessed to identify original studies reporting direct experimental exposure to microplastic particles and biological responses in marine microalgae. Based on these criteria, 43 experimental studies were included in the comparative synthesis. Experimental information extracted from these studies included microalgal species, polymer type, particle size, exposure concentration and duration, biological endpoints, and main ecotoxicological outcomes (Supplementary Table S2). The complete selection and analytical workflow are summarized in Fig. 1.

### Quantitative literature analysis and evidence mapping

Quantitative evidence mapping was conducted using the bibliometrix package (R statistical software) to support the interpretation of patterns within the 59 selected publications (Aria and Cuccurullo 2017). The biblioshiny web interface was utilized to visualize data distributions and generate graphical outputs, facilitating the synthesis of information related to publication patterns and study characteristics. Journal-level information and publication patterns were used only to contextualize the experimental ecotoxicological evidence. These analyses were applied exclusively as a complementary tool to support the interpretation of experimental ecotoxicological evidence, rather than as a primary objective of the study.

This approach was complemented by integrating qualitative and quantitative information from the two analytical datasets, enabling a deeper interpretation of research patterns and variability in experimental design (Zupic and Čater 2015). Bibliometric and descriptive information, including year of publication and geographic origin, was analyzed across the 59 selected publications. Comparative ecotoxicological data, including microalgal taxonomic classification, polymer type and particle size, exposure conditions, and evaluated biological endpoints, were systematically extracted from the 43 experimental studies included in the comparative synthesis (Supplementary Table S2).

### Classification of endpoints and data synthesis

The endpoint classification was designed to align the literature synthesis with commonly used ecotoxicological response categories applied in laboratory toxicity tests with phytoplankton and primary producers. To facilitate comparative analysis, endpoints reported in the 43 experimental studies were grouped into six functional categories based on similarities in biological focus: (1) growth/population parameters; (2) cellular stress and damage indicators; (3) structural/interaction parameters; (4) photosynthetic physiology; (5) photosynthetic biochemistry; and (6) biochemical/metabolic responses. The grouping was developed for the present synthesis and informed by established concepts in microplastic ecotoxicology (Wright et al. 2013; Hartmann et al. 2019). Individual studies could contribute to more than one functional category when multiple endpoints were evaluated. This classification enabled the identification of frequently assessed endpoints and underexplored areas within the literature. The synthesized findings provided the framework for the subsequent Results and Discussion sections, emphasizing research trends, methodological heterogeneity, and existing knowledge gaps concerning the impacts of microplastic pollution on marine microalgae.

## Results and discussion

### Selected publications

Among the 59 selected publications, 51 were indexed as original research articles (86.4%) and 8 as review articles (13.6%) in the bibliographic dataset (Table 1). After removing duplicate records based on Digital Object Identifiers (DOIs), titles, and author names, titles, abstracts, and keywords were screened to determine their relevance to the research scope. Publications that did not specifically address interactions between MPs and marine microalgae were excluded. After the full screening procedure, a final dataset of 59 publications addressing microplastic–marine microalgae interactions was obtained (Fig. 1). Of these, 43 original experimental studies met the specific eligibility criteria for the comparative ecotoxicological synthesis. The complete list of 59 selected publications used in the bibliometric characterization and evidence mapping is provided in Supplementary Table S1, whereas the comparative experimental data for the 43 studies are summarized in Supplementary Table S2.

### Temporal trends and characteristics of selected studies

Although the search parameters covered the period from 1970 to August 2025, no eligible publications were identified before 2016, indicating that the absence of earlier publications reflects the relatively recent emergence of this specific research field rather than a limitation of the search period. This temporal pattern is consistent with the development of microplastic research involving microalgae more broadly, as early experimental studies addressing both marine and freshwater microalgae were published in 2016 (Sjollema et al. 2016; Lagarde et al. 2016). In contrast, experimental evidence of microplastic interactions with other marine taxa had already been reported earlier, including studies with mussels and zooplankton (Browne et al. 2008; Cole et al. 2013). Thus, the emergence of studies focusing specifically on microalgae appears to represent a later phase in the development of aquatic microplastic ecotoxicology.

The selected publications were distributed across 15 scientific journals and involved a total of 303 authors, reflecting the growing engagement of the scientific community in investigating the ecological effects of MPs on marine primary producers.

**Table 1:** General bibliometric characteristics of the 59 selected publications addressing microplastic–marine microalgae interactions

Parameter	Value
Publication period	2016 to 2025
Average annual growth rate (%)	24.16
Number of journals	15
Number of journals in Environmental Science category	11
Number of journals in the Toxicology category	4
Number of publications analyzed	59
Number of original research articles	51
Publications indexed as reviews	8
Average age of publications (years)	2.90
Total number of publication references	2152
Total number of authors	303
Publications with international co-authorship (%)	11.86

**Fig. 2:**
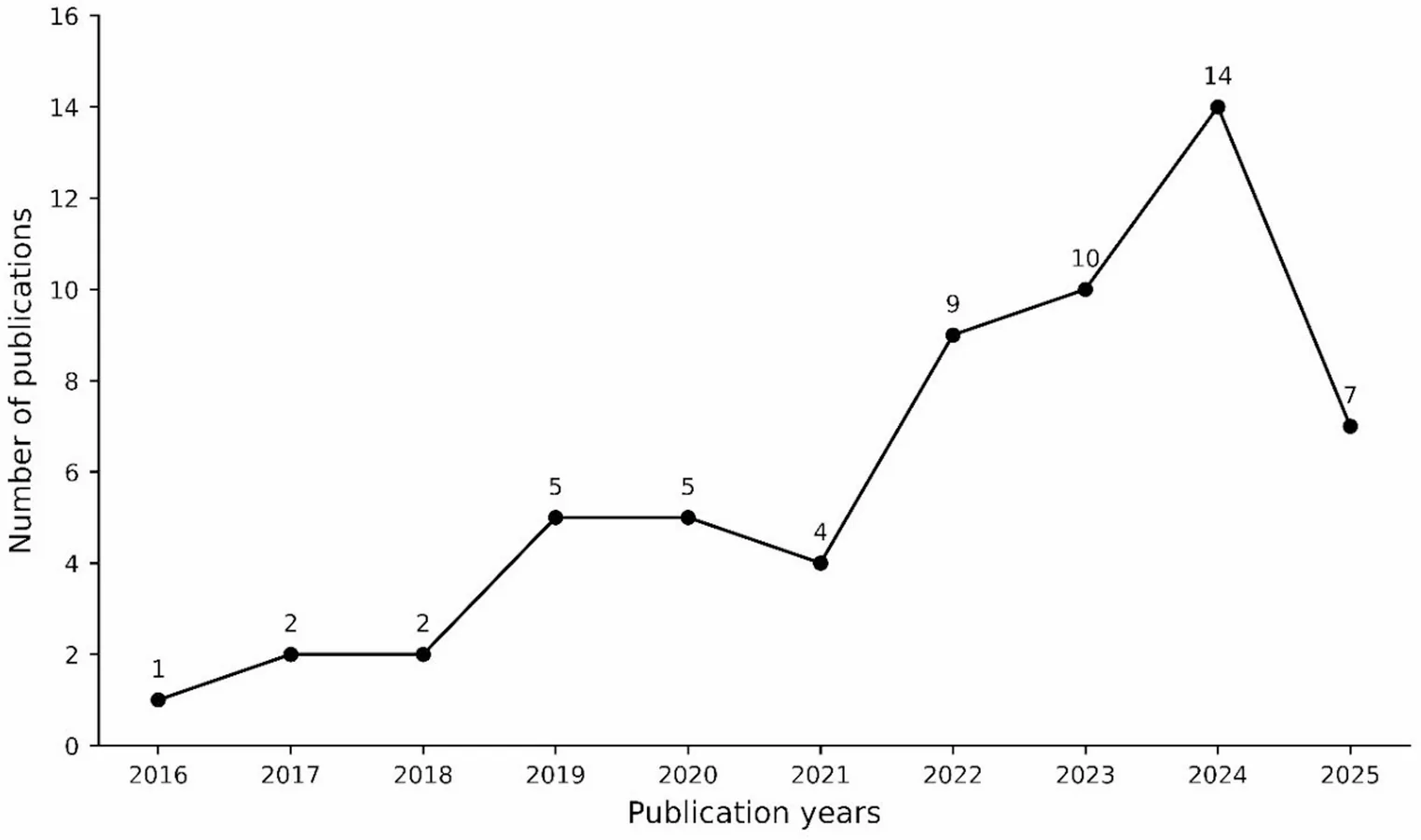
Temporal distribution of the 59 selected scientific publications addressing microplastic–marine microalgae interactions between 2016 and August 2025

The temporal distribution of publications reveals a marked increase in research activity over the past decade (Fig. 2). The number of publications rose from a single publication in 2016 to 14 in 2024, demonstrating a consistent upward trend. This pattern reflects the increasing recognition of MPs as emerging contaminants in marine environments and the growing interest in understanding their ecological impacts on primary producers. This increase indicates a rapid expansion of research interest in microplastic toxicity to marine microalgae over the last decade, consistent with the broader acceleration of microplastic research reported in bibliometric assessments (Li et al. 2022). Nevertheless, the relatively small absolute number of publications identified here indicates that this specific ecotoxicological field remains at an early stage of development.

The limited number of studies focusing specifically on marine microalgae should also be considered within the broader taxonomic context of microplastic ecotoxicology. While the evidence identified here for marine microalgae remains relatively limited, microplastic effects have already been quantitatively synthesized across a range of fish and aquatic invertebrate taxa through meta-analytical approaches (Foley et al. 2018). This contrast further highlights the comparatively early stage of the evidence base concerning marine primary producers.

The predominance of multi-authored studies, with no single-author publications identified, highlights the collaborative nature of research in this field. However, only 11.86% of the publications involved international co-authorship, suggesting that research efforts remain largely concentrated within national research groups. This limited international collaboration may contribute to methodological heterogeneity across studies, particularly in relation to experimental design, particle characteristics, and exposure conditions.

Overall, the rapid increase in publication output, combined with the expansion of collaborative research networks, indicates that this field is undergoing accelerated development. In addition, the growing number of studies suggests a shift toward more detailed investigations of physiological and biochemical responses, reinforcing the importance of ecotoxicological approaches for evaluating the environmental risks associated with microplastic contamination.

### Spatial distribution of publications

The analysis identified contributions from 16 countries among the 59 selected publications addressing microplastic–marine microalgae interactions. China accounted for the largest share of publications (54.24%), followed by Spain and South Korea (6.78% each), and Portugal (5.08%). France, Italy, Belgium, and Brazil each contributed 3.39% of the publications, while Argentina, the Czech Republic, Egypt, India, Malaysia, the Netherlands, Poland, and the United States published one article each during this period. This distribution is consistent with global trends reported for microplastic research, which consistently identified China as a leading contributor to scientific output in aquatic ecotoxicology (Oliveira et al. 2020; Akinpelu et al. 2022; Gao et al. 2022). This dominance may be associated with substantial increases in research funding, notably through the expansion of the National Natural Science Foundation of China since 2015 (Yang 2016), as well as national prioritization of research addressing plastic pollution.

Despite the global nature of the plastic crisis, international collaboration remains relatively limited; only seven of the 59 analyzed **publications** involved Multiple-Country Participation (MCP) (Table 2). Among these, China spearheaded three publications in partnership with authors from Indonesia, Australia, Malaysia, South Korea, Iran, the Czech Republic, Pakistan, and Argentina. Remaining collaborations included efforts between Spain and France, two by France with Spain and Australia, and one by Egypt with Saudi Arabia. This low MCP rate suggests that research on microplastic–microalgae interactions is still largely conducted within national boundaries, highlighting a significant opportunity for cross-border initiatives that could facilitate methodological standardization across studies.

**Table 2:** Spatial distribution of the selected publications addressing microplastic–marine microalgae interactions (*n* = 59)

Classification	Country	Articles	SCP	MCP
1	China	32	29	3
2	Spain	4	3	1
3	South Korea	4	4	0
4	Portugal	3	3	0
5	France	2	0	2
6	Italy	2	2	0
7	Belgium	2	2	0
8	Brazil	2	2	0
9	Argentina	1	1	0
10	Czech Republic	1	1	0
11	Egypt	1	0	1
12	India	1	1	0
13	Malaysia	1	1	0
14	Netherlands	1	1	0
15	Poland	1	1	0
16	United States	1	1	0
Total		**59**	**52**	**7**

SCP = number of publications from a single country. MCP = number of publications from multiple countries. Total = number of publications

### Distribution of publications across environmental and ecotoxicological journals

The distribution of publications across scientific journals indicates that research on interactions between microplastics and marine microalgae is concentrated within a relatively small group of environmental and ecotoxicological journals (Table 3). Environmental Pollution and Science of the Total Environment account for the highest number of publications, followed by journals focused on aquatic toxicology and environmental contamination. This pattern reflects the growing scientific interest in understanding the ecological consequences of microplastic exposure in aquatic primary producers and highlights the relevance of this topic within the broader fields of environmental pollution and ecotoxicology. The concentration of publications in journals addressing contaminant dynamics, aquatic toxicology, and ecosystem health also illustrates the increasing integration of microplastic research into ecotoxicological evaluation frameworks. Overall, this distribution reinforces the recognition of MPs as emerging contaminants capable of affecting key biological processes in marine primary producers and potentially influencing ecological functioning in aquatic ecosystems.

**Table 3:** Distribution of the selected publications across journals addressing microplastic–marine microalgae interactions (*n* = 59)

Classification	Journal	Articles
1	*Environmental Pollution*	14
2	*Science of the Total Environment*	10
3	*Aquatic Toxicology*	7
4	*Marine Environmental Research*	6
5	*Journal of Hazardous Materials*	5
6	*Chemosphere*	4
7	*Water Research*	3
8	*Ecotoxicology and Environmental Safety*	2
9	*Marine Pollution Bulletin*	2
10	*Environmental Chemistry Letters*	1
11	*Environmental Research*	1
12	*Environmental Science and Pollution Research*	1
13	*Frontiers in Toxicology*	1
14	*Regional Studies in Marine Science*	1
15	*Environments*	1

### Key findings from influential publications

The scientific literature has substantially advanced the understanding of interactions between microplastics (MPs) and marine primary producers, suggesting that microplastic exposure may induce a wide range of physiological and biochemical responses in microalgae. Across experimental studies, converging evidence suggests that growth inhibition and impairment of photosynthetic performance are among the most consistent responses, reflecting the sensitivity of primary producers to particle-induced stress.

These toxic effects may arise from multiple, often interconnected mechanisms, including both physical and biochemical pathways. Such responses are often mediated by oxidative stress pathways, where the overproduction of reactive oxygen species (ROS) leads to lipid peroxidation, membrane damage, and impairment of cellular functions. This framework contributes to understanding potential pathways through which microplastic exposure may translate into functional impairments at the cellular level, distinguishing this synthesis from predominantly descriptive approaches. Direct physical interactions between MPs and algal cell surfaces can lead to mechanical interference, as demonstrated in the diatom *Skeletonema costatum* (Zhang et al. 2017). Microplastic–microalgae interactions can also promote species-specific hetero-aggregation, influencing particle dynamics in the water column (Long et al. 2017). Such interactions can modify light availability and nutrient exchange, indirectly affecting microalgal metabolism and growth.

Beyond physical interactions, MPs may also act as vectors for chemical contaminants, thereby modifying the toxicity of co-occurring substances. Prata et al. (2018) demonstrated that the presence of MPs can alter the toxicity of pharmaceutical compounds such as procainamide and doxycycline. These combined effects are influenced by particle size and the relative dimensions of microplastics and algal cells, which can determine the extent of cell–particle interactions (Chae et al. 2019). Complementary evidence from studies focusing on particle-fate processes also highlights the role of algal extracellular polymeric substances (EPS) in mediating aggregation, potentially affecting the environmental fate and bioavailability of MPs (Lim et al. 2024; Sioen et al. 2024).

In addition to mechanistic insights, previous reviews (Prata et al. 2019; Nava and Leoni 2021) emphasize that microplastic exposure may disrupt key ecological processes, including population dynamics, nutrient cycling, and energy transfer within marine ecosystems. Reported effects include reductions in chlorophyll content, alterations in photosynthetic activity, and morphological changes, all of which may compromise primary productivity. Importantly, these responses are not uniform across taxa, indicating that species-specific traits play a critical role in modulating microplastic toxicity.

Collectively, the available evidence indicates that microplastic toxicity appears to result from a combination of physical and biochemical interactions. The most influential publications in this field, based on citation metrics, are summarized in Table 4.

**Table 4:** Ten most cited publications identified in the bibliometric dataset (*n* = 59)

References	Title	Journal	Total Citations	Average Citations per Year		
Zhang et al. (2017)	Toxic effects of microplastic on marine microalgae *Skeletonema costatum*: Interactions between microplastic and algae	*Environmental Pollution*	476		59.5	
Sjollema et al. (2016)	Do plastic particles affect microalgal photosynthesis and growth?	*Aquatic Toxicology*	410	45.5		
Prata et al. (2019)	Effects of microplastics on microalgae populations: A critical review	*Science of the Total Environment*	232	38.6		
*Long* et al. (2017)	Interactions between polystyrene microplastics and marine phytoplankton lead to species-specific hetero-aggregation	*Environmental Pollution*	225	28.1		
*Prata* et al. (2018)	Influence of microplastics on the toxicity of the pharmaceuticals procainamide and doxycycline on the marine microalgae *Tetraselmis chuii*	*Aquatic Toxicology*	215	30.7		
*Nava and Leoni* (2021)	A critical review of interactions between microplastics, microalgae, and aquatic ecosystem function	*Water Research*	161	40.2		
*Cunha* et al. (2019)	Marine vs. freshwater microalgae exopolymers as biosolutions to microplastics pollution	*Environmental Pollution*	113	18.8		
*Chae* et al. (2019)	Effects of micro-sized polyethylene spheres on the marine microalgae *Dunaliella salina*: Focusing on the algal cell to plastic particle size ratio	*Aquatic Toxicology*	102	17.0		
*Zhao* et al. (2019)	The interactions between micro polyvinylchloride (mPVC) and marine dinoflagellate *Karenia mikimotoi*: The inhibition of growth, chlorophyll and photosynthetic efficiency	*Environmental Pollution*	86	14.3		
Gambardella et al. (2018)	Ecotoxicological effects of polystyrene microbeads in a battery of marine organisms belonging to different trophic levels	*Marine Environmental Research*	84	12.0		

### Polymeric classes of microplastics studied

**Fig. 3:**
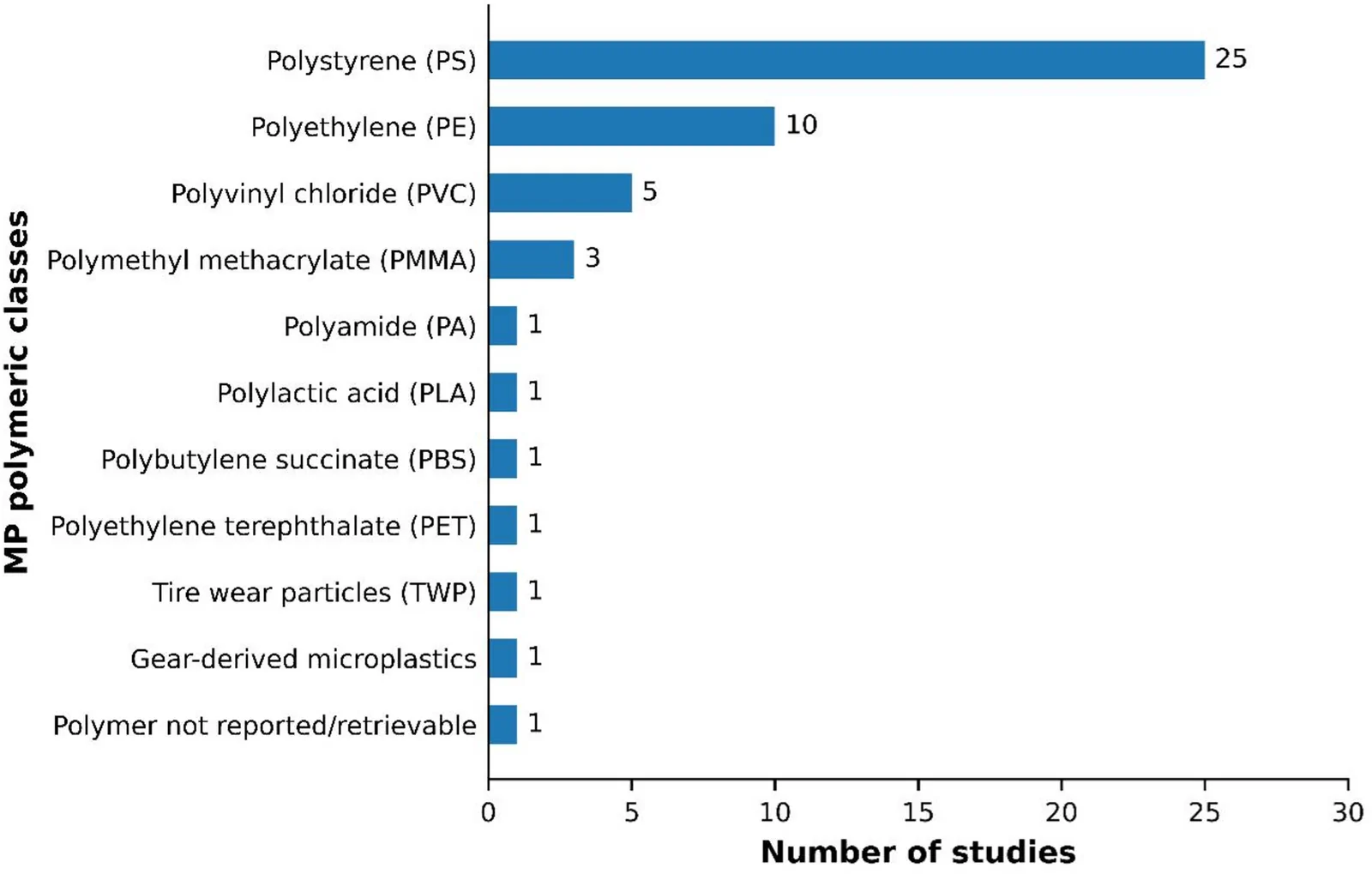
Polymer types investigated among the 43 experimental studies evaluating microplastic effects on marine microalgae. Because individual studies could investigate more than one polymer type, frequencies are not mutually exclusive

The comparative ecotoxicological synthesis was based on the 43 experimental studies that met the specific eligibility criteria described in Sect.  2.2 (Supplementary Table S2). Among the polymer types investigated, polystyrene (PS) and polyethylene (PE) were the most frequently examined, being reported in 25 and 10 studies, respectively (Fig. 3). These were followed by polyvinyl chloride (PVC; 5 studies) and polymethyl methacrylate (PMMA; 3 studies). Polyamide (PA), polylactic acid (PLA), polybutylene succinate (PBS), polyethylene terephthalate (PET), tire wear particles (TWP), and gear-derived microplastics were each investigated in one study, while the polymer type was not reported (NR) in one study. Because individual studies could investigate more than one polymer type, these frequencies are not mutually exclusive. The polymer classes represented in the experimental literature include several polymers commonly reported in marine plastic pollution; however, their relative representation in laboratory studies does not necessarily reflect their environmental abundance (Andrady 2017; Nava and Leoni 2021).

The overrepresentation of PS in toxicological assays—despite PE and PP often being found in higher concentrations in surface waters—may partly reflect the widespread use and commercial availability of standardized PS spheres and microbeads in laboratory assays. The comparatively limited representation of other polymer classes indicates that the composition used in laboratory studies may not fully reflect the diversity and relative abundance of polymers occurring in natural marine environments. This discrepancy has important implications for ecotoxicological interpretation and for the extrapolation of laboratory findings to environmentally realistic exposure scenarios.

### Marine microalgae studied

The marine microalgae investigated across the 43 experimental studies included in the comparative ecotoxicological synthesis were distributed among eight major taxonomic groups (Table 5). Bacillariophyta (diatoms) represented the largest proportion, being investigated in 22 studies (51.2%), followed by Chlorophyta in 10 studies (23.3%) and Dinophyta in 8 studies (18.6%). Haptophyta was represented in 4 studies (9.3%), while Ochrophyta (Raphidophyceae) and Cryptophyta were each represented in 2 studies (4.7%), and Cyanobacteria and Rhodophyta were each represented in 1 study (2.3%). Because some studies investigated species belonging to more than one taxonomic group, these percentages are not mutually exclusive. This distribution reflects the ecological prominence of diatoms and dinoflagellates in marine ecosystems and underscores their established role as model organisms in ecotoxicological research. Their frequent selection is largely due to their fundamental contributions to global primary productivity, nutrient cycling, and bloom dynamics (Oliveira et al. 2020; Naselli-Flores 2023).

The high representation of *Bacillariophyta* and the recurrent representation of *Dinophyta* and *Chlorophyta* reflects the research focus on the most abundant and diverse phytoplankton groups in coastal and oceanic waters. However, the relatively low percentage of other taxa, such as *Haptophyta*, suggests a potential knowledge gap in understanding the impacts of MPs on other ecologically significant groups, such as coccolithophores, which play a critical role in the marine calcium carbonate cycle. Expanding the taxonomic diversity in future experimental designs will be vital for a more holistic assessment of microplastic-induced shifts in marine community structures.

**Table 5:** Taxonomic groups and marine microalgal species investigated in the 43 experimental studies included in the comparative ecotoxicological synthesis

Taxonomic group	Studies, *n* (%)	Species represented
Bacillariophyta	22 (51.2)	*Amphora coffeaeformis*; *Chaetoceros calcitrans*; *Chaetoceros curvisetus*; *Chaetoceros gracilis*; *Chaetoceros neogracile*; *Nitzschia closterium* f. *minutissima*; *Phaeodactylum tricornutum*; *Skeletonema costatum*; *Thalassiosira pseudonana*; *Thalassiosira weissflogii*
Chlorophyta	10 (23.3)	*Chlorella vulgaris*; *Dunaliella salina*; *Dunaliella tertiolecta*; *Platymonas helgolandica*; *Tetraselmis chuii*; *Tetraselmis* sp.
Dinophyta	8 (18.6)	*Akashiwo sanguinea*; *Alexandrium tamarense*; *Gymnodinium aeruginosum*; *Gymnodinium* sp.; *Heterocapsa triquetra*; *Karenia mikimotoi*; *Prorocentrum donghaiense*; *Prorocentrum micans*; *Prorocentrum minimum*
Haptophyta	4 (9.3)	*Isochrysis galbana*; *Tisochrysis lutea*
Ochrophyta (Raphidophyceae)	2 (4.7)	*Heterosigma akashiwo*
Cryptophyta	2 (4.7)	*Rhodomonas lens*; *Rhodomonas salina*
Cyanobacteria	1 (2.3)	*Gloeocapsa* sp.
Rhodophyta	1 (2.3)	*Porphyridium purpureum*

Note: Individual studies could include species from more than one taxonomic group; therefore, frequencies and percentages are not mutually exclusive

### Endpoints studied

**Fig. 4:**
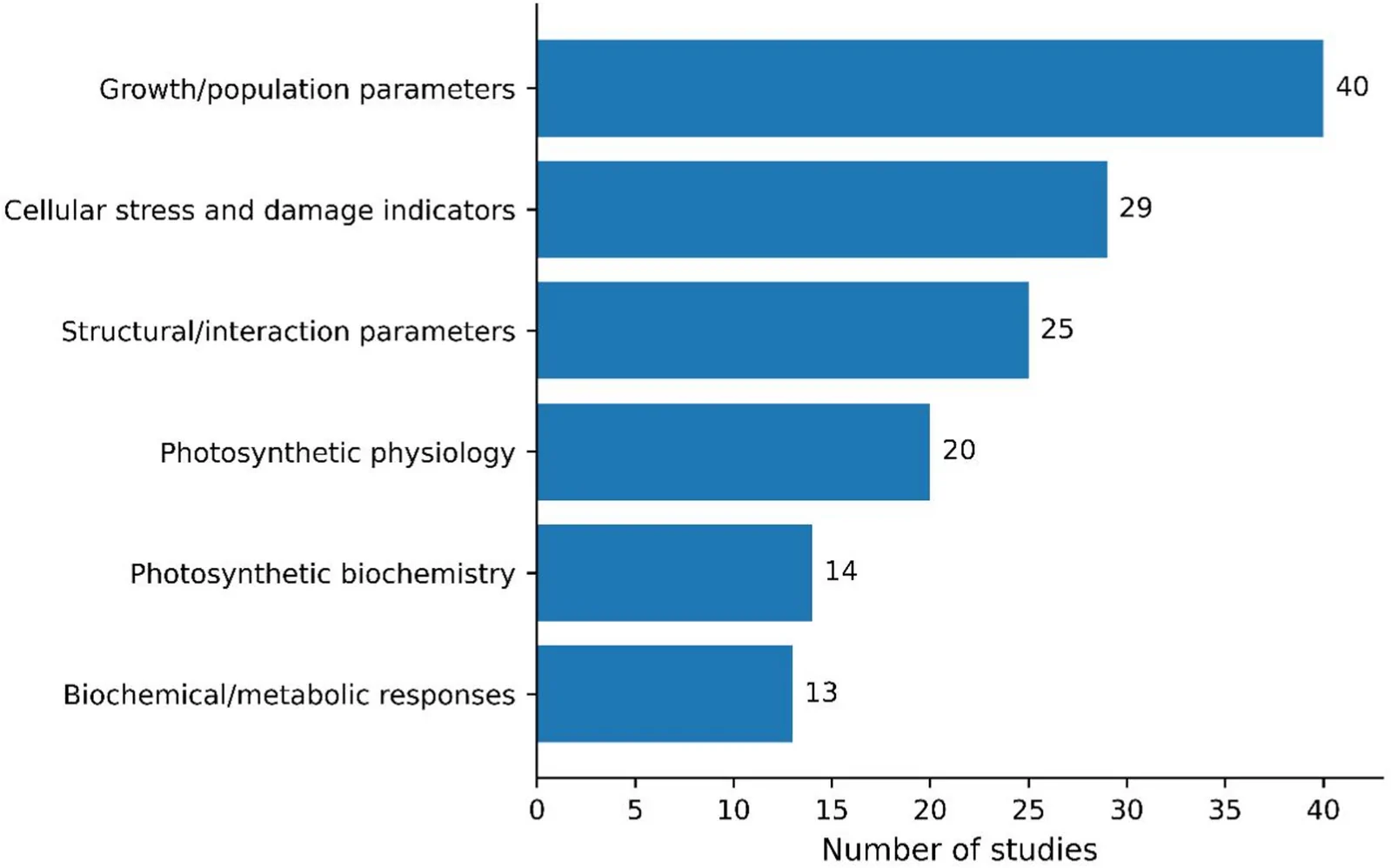
Functional categories of endpoints evaluated in the 43 experimental studies on microplastic effects in marine microalgae. Individual studies could evaluate endpoints belonging to more than one functional category; therefore, frequencies are not mutually exclusive

Understanding which biological endpoints are most frequently evaluated in experimental microalgal toxicity studies is essential for improving ecotoxicological test design and ecotoxicological evaluation. Therefore, the endpoints reported in the selected studies were systematically categorized to identify dominant response parameters and underexplored indicators of microplastic toxicity. The endpoints reported across the 43 experimental studies included in the comparative ecotoxicological synthesis were grouped into six functional categories based on biological response and analytical focus (Fig. 4): (1) growth/population parameters; (2) cellular stress and damage indicators; (3) structural/interaction parameters; (4) photosynthetic physiology; (5) photosynthetic biochemistry; and (6) biochemical/metabolic responses.

Growth/population parameters represented the most frequently investigated endpoint group, addressed in 40 studies (Fig. 4). Within this category, growth inhibition, growth rate, cell density, and cell viability were among the responses evaluated across studies. The data suggest that particle size may influence the mechanisms of toxicity: smaller particles may exhibit greater toxicity due to enhanced surface interactions (Chae et al. 2019; Wang et al. 2024), while larger particles may induce growth inhibition through shading and other physical interference mechanisms (Zhang et al. 2017; Guo et al. 2020).

Cellular stress and damage indicators were evaluated in 29 studies (Fig. 4). Oxidative stress was among the most frequently investigated responses within this category, although the magnitude and direction of the response varied according to MP concentration and experimental conditions. Furthermore, research has identified damage to intracellular organelles and reductions in esterase activity and neutral lipid production (Seoane et al. 2019; Wang et al. 2024). The increasing focus on oxidative stress in recent years (i.e., Wang et al. 2025a, Wang et al. 2025c; Zhao et al. 2025) reflects a shift toward understanding the sub-lethal biochemical pathways of plastic toxicity.

Photosynthetic physiology was evaluated in 20 studies (Fig. 4). Effects on photosynthetic rate, photosynthetic efficiency, photochemistry, and chlorophyll fluorescence were investigated across these studies, with responses varying among experimental conditions. Studies by Prata et al. (2018), Zhao et al. (2019), and Wang et al. (2020) demonstrated that microplastic exposure may impair microalgal photosynthesis, resulting in reduced chlorophyll content and photosynthetic efficiency. Zhao et al. (2019) reported that micro-PVC MPs affected chlorophyll content, photosynthetic efficiency, and cell density in the dinoflagellate *Karenia mikimotoi*. In contrast, Chae et al. (2019) observed an increase in chlorophyll content when microalgae were exposed to high MPs concentrations.

Photosynthetic biochemistry was assessed in 14 studies (Fig. 4), whereas structural/interaction parameters were evaluated in 25 studies. Biochemical/metabolic responses were also evaluated in 13 studies. Guo et al. (2020) reported alterations in lipid and fatty acid composition in *Phaeodactylum tricornutum*. These results raise important questions regarding the impacts of MPs on microalgal biomolecule production and the transfer of essential lipid compounds across trophic chains, highlighting the need for further investigation.

Complementary evidence on aggregate formation indicates that interactions between MPs and microalgae may induce shading effects, potentially reducing photosynthetic efficiency and microalgal growth (Jin et al. 2024). Shading effects have also been investigated as a potential mechanism through which MPs may affect microalgae, with studies reporting growth inhibition, morphological alterations, membrane damage, and reductions in photosynthetic activity (Chae et al. 2019; Wang et al. 2020; Li et al. 2024). Additionally, MPs were shown to modulate energy metabolism by decreasing lipid-producing cells (Seoane et al. 2019).

In addition to particle size and polymer type, environmental transformation of MPs may substantially modify their interactions with microalgae. Aging and weathering can alter particle surface chemistry, charge, roughness, and functional groups, thereby affecting aggregation, contaminant adsorption, additive leaching, and biological interactions. Similarly, adsorption of dissolved organic matter and algal extracellular polymeric substances may promote eco-corona formation, modifying particle stability and cell–particle interactions. Experimental evidence with marine microalgae indicates that EPS-mediated eco-corona formation can reduce the toxicity of polystyrene nanoplastics by decreasing particle–cell interactions and oxidative stress (Natarajan et al. 2020). Biofilm development on plastic surfaces may further modify particle density, transport, and biological availability. Conversely, weathering may enhance the release of polymer-associated additives or degradation products, potentially increasing chemical contributions to toxicity (Luo et al. 2022). These processes demonstrate that MPs should not be regarded as static particles and may partly explain differences among experimental studies using pristine versus environmentally transformed plastics. However, aging, eco-corona formation, biofilm development, and additive leaching remain insufficiently represented in experimental studies with marine microalgae, limiting their incorporation into current ecotoxicological assessment frameworks.

Beyond the experimental studies included in the comparative synthesis, Leite et al. (2022) integrated field-based analyses, including the identification and quantification of MPs (sizes 1.01 to 4.75 mm) in global surface waters. This study assessed the role of floating plastics as vectors for toxin transfer to three benthic dinoflagellate species: *Gambierdiscus* spp., *Ostreopsis* cf. *ovata*, and *Prorocentrum lima*. Distribution analysis indicated elevated relative risks of plastic colonization in the Mediterranean Sea and along subtropical and temperate western ocean margins, such as the eastern coasts of North America and Asia, and to a lesser extent, in southern Brazil and Australia (Leite et al. 2022).

Overall, the effects of MPs—whether acting alone or synergistically with other contaminants—on growth inhibition, chlorophyll *a* content, cell density, oxidative stress, and photosynthetic efficiency are the most frequently investigated and represent the most established parameters in the available literature. In contrast, critical endpoints such as sinking rate, shading effects, aggregate formation, DNA damage, and relative electron transfer remain significantly under-explored. Furthermore, several studies report transient effects, where an initial period of physiological vulnerability is followed by adaptive responses leading to physiological recovery (Prata et al. 2019). These patterns may reflect intrinsic organismal resilience or shifts in MPs bioavailability within exposure media over time. These findings highlight the importance of quantifying MPs bioavailability and distribution within experimental frameworks. Achieving accurate estimates of the effective exposure concentrations is vital for bridging the gap between controlled laboratory settings and the complex dynamics of marine environments (Long et al. 2017).

From an ecotoxicological perspective, the current evidence does not yet support a definitive ranking of endpoints according to sensitivity, because differences in species, particle characteristics, exposure concentrations, duration, and analytical methods limit direct comparisons among studies. Nevertheless, growth/population parameters and photosynthetic responses appear particularly informative for future comparative testing because they are biologically relevant, widely evaluated across the available studies, and directly related to microalgal performance. Growth-related endpoints also offer the advantage of established use in standardized algal toxicity testing, facilitating comparison among experiments (OECD 2011). Photosynthetic endpoints may provide complementary information on sublethal physiological impairment, whereas oxidative-stress and other biochemical biomarkers can contribute mechanistic information but currently show greater methodological heterogeneity across studies. Therefore, future standardized approaches could benefit from combining a robust apical endpoint, such as growth rate or growth inhibition, with complementary physiological endpoints related to photosynthetic performance, while biochemical and molecular responses may be particularly useful for mechanistic interpretation.

Despite the rapid increase in experimental studies addressing microplastic toxicity in marine microalgae, the environmental relevance of the reported effects remains difficult to establish. The experimental dataset compiled here reveals substantial variability in particle size, polymer type, exposure concentration, and exposure duration across studies (Supplementary Table S2). Many experiments were designed primarily to investigate mechanisms of toxicity and employed controlled exposure scenarios and concentrations that may exceed those typically encountered in natural marine environments. Effects observed under such conditions are valuable for identifying potential modes of action but should not be directly interpreted as evidence of ecological effects under field conditions. Indeed, studies conducted with environmentally relevant concentrations and particle-size distributions have reported limited or no effects on microalgal growth under the tested conditions (Niu et al. 2021). Consequently, the available evidence currently provides stronger support for identifying potential toxicity mechanisms than for quantitatively predicting ecological risk. Future studies combining mechanistically informative laboratory exposures with environmentally representative particle characteristics, concentration ranges, and longer exposure periods are therefore needed to improve environmental extrapolation.

Because marine microalgae constitute the base of aquatic food webs, alterations in their physiology and growth may potentially influence trophic transfer processes and the movement of MPs through marine food chains. Overall, the available evidence indicates that while several key physiological endpoints of microplastic toxicity in marine microalgae are increasingly well documented, important knowledge gaps remain regarding environmentally realistic exposure scenarios and underexplored biological responses. Collectively, these findings suggest that microplastic exposure may influence key physiological processes that regulate marine primary productivity, potentially affecting energy transfer and trophic dynamics within aquatic ecosystems.

By identifying the endpoints for which evidence is most consistent, together with the experimental conditions and biological responses that remain insufficiently explored, this review provides a basis for prioritizing future ecotoxicological research on marine microalgae. Importantly, rather than establishing a direct link between laboratory responses and effects under natural conditions, our synthesis identifies the experimental limitations that currently constrain such extrapolation and highlights the need for environmentally realistic exposure scenarios to improve the ecological interpretation of microplastic toxicity.

## Conclusion

This review provides a comparative synthesis of the experimental evidence on microplastic effects in marine microalgae, a rapidly expanding but still methodologically heterogeneous field. Across the available studies, growth/population responses, cellular stress and damage, and photosynthetic alterations emerge as the most frequently investigated biological responses. However, differences in polymer type, particle size, exposure concentration and duration, and experimental design limit direct comparisons among studies and constrain extrapolation from laboratory responses to natural marine environments.

Important knowledge gaps remain regarding environmentally relevant processes, including microplastic–microalgae aggregation, particle sinking dynamics, DNA damage, environmentally transformed particles, and long-term exposure under realistic concentrations. Future research should therefore prioritize standardized experimental approaches, environmentally representative particles and exposure scenarios, and greater taxonomic and endpoint diversity.

By systematically comparing experimental conditions and biological responses across the available evidence, this review provides an ecotoxicological framework for distinguishing well-investigated responses from underexplored endpoints and for identifying the methodological limitations that currently constrain environmental interpretation. Rather than implying direct ecological effects from laboratory responses, the synthesis defines priorities for improving the comparability, environmental relevance, and ecological interpretation of future microplastic toxicity studies with marine primary producers.

## Supplementary Information

Below is the link to the electronic supplementary material.

Supplementary Material 1

## Data Availability

All data generated or analyzed during this study are included in this article and its supplementary information files. The dataset used for bibliometric characterization and evidence mapping is provided in Supplementary Table S1, the comparative experimental dataset in Supplementary Table S2, and the complete database-specific search strategies in Supplementary Table S3.
